# Common microRNA–mRNA Interactions in Different Newcastle Disease Virus-Infected Chicken Embryonic Visceral Tissues

**DOI:** 10.3390/ijms19051291

**Published:** 2018-04-25

**Authors:** Yan-Qing Jia, Xing-Long Wang, Xiang-Wei Wang, Chuan-Qi Yan, Chang-Jie Lv, Xiao-Qin Li, Zhi-Li Chu, Fathalrhman Eisa Addoma Adam, Sa Xiao, Shu-Xia Zhang, Zeng-Qi Yang

**Affiliations:** 1 College of Veterinary Medicine, Northwest A&F University, Yangling 712100, China; yqjia1987@163.com (Y.-Q.J.); wxlong@nwsuaf.edu.cn (X.-L.W.); wxw2014hc@163.com (X.-W.W.); ycq875935701@163.com (C.-Q.Y.); lcjandjt@163.com (C.-J.L.); L18584890329@163.com (X.-Q.L.); zhilichu@nwafu.edu.cn (Z.-L.C.); fathalrhmanaddoma@gmail.com (F.E.A.A.); saxiao@nwafu.edu.cn (S.X.); zhangshuxia316@163.com (S.-X.Z.); 2 Department of Preventive Medicine and Public Health, Faculty of Veterinary Science, University of Nyala, P.O. Box, 155 Nyala, Sudan

**Keywords:** chicken embryo, Newcastle disease virus, RNA-Seq, microRNA, mRNA

## Abstract

To investigate the roles and explore the altered expression of microRNAs (miRNAs) and mRNAs in chicken embryos in response to Newcastle disease virus (NDV) infection, deep sequencing was performed. Then, a conjoint analysis of small RNA-seq and mRNA-seq was performed to screen interactional miRNA–mRNA pairs during NDV infection. In total, 15 and 17 up- and downregulated miRNAs were identified that potentially targeted 4279 and 6080 mRNAs in NDV-infected chicken embryonic tissues, respectively; in addition, 595 upregulated and 480 downregulated mRNAs were identified. The conjoint analysis of the obtained data identified 1069 miRNA–mRNA pairs. Among these pairs, 130 pairs were related to immune or inflammatory responses. The relationship between gga-miR-203a and its target transglutaminase 2 (TGM2) was confirmed using a dual-luciferase reporter system and a real time quantitative polymerase chain reaction (RT-qPCR) assay. Overall, the discovery of miRNAs, mRNAs, and their potential pairing relationships, which may be involved in the regulation of NDV infection, will facilitate our understanding of the complex regulatory relationship between the host and the virus.

## 1. Introduction

Newcastle disease virus (NDV), which is the causative agent of Newcastle disease, remains prevalent worldwide since its first isolation in 1935 [1,2]. Because of their high genetic variability, NDVs have shown both increase in virulence and capability to cross the host barrier, including transmission from birds to mammals, and current statistics show that more than 250 different species can be infected by NDV [2,3,4]. The high mutation rate of NDV reduces vaccine efficiency, may consequently lead to great economic losses to the global poultry industry, and is a major obstacle to ND prevention and control [3,5,6]. 

Disease from NDV infection results from the interaction between the virus and the host’s immune response, and a constant struggle exists between the virus and host immune system. The pathogenicity of a virus is determined not only by its characteristics but also by the host immune response [7,8,9]. Therefore, a comprehensive overview of the molecular mechanisms of the interaction between the host and NDV infection may increase our understanding of host stress immunity and assist in the identification of a potential target for the prevention and treatment of NDV infection. 

Molecular biology has expanded our knowledge of the pathophysiology of viral infection, particularly after the advent of genome-wide microRNA (miRNA) and mRNA expression profiling using deep sequencing, which has provided new insight into the interactions between host and viral infection [10,11,12,13]. miRNAs are important cellular regulatory factors that regulate post-transcriptional gene expression by degrading their transcription or blocking translation [14,15,16,17,18]. The roles of miRNAs in the regulation of virus infection, such as Avian influenza virus (AIV), Infectious Bursal disease virus (IBDV), Marek’s disease virus (MDV), Hepatitis C virus (HCV), etc., have all been previously identified [19,20,21,22]. In addition to affecting gene expression in the host, miRNAs can bind viral genes to regulate viral replication [23,24]. For example, miR-485 increases viral proliferation by degrading the host’s Retinoic acid inducible-gene I (RIG-I) gene during NDV or low-dose AIV infections, whereas it inhibits AIV replication by targeting the PB1 gene when the viral loads are increased [25]. miR-122, which is a liver-specific miRNA, is essential for HCV replication [24,26]. However, information regarding the role of miRNAs in NDV infection in chickens is limited. 

The limited reports of the mRNA transcriptome in tissues or cells after NDV infection have revealed useful information. mRNA transcript profiles obtained from NDV/AIV-infected embryonic fibroblasts (CEFs) have provided a comprehensive picture of gene expression in infected cells and revealed the dynamic host response to NDV/AIV, including a delayed type I interferon-stimulated gene expression [27]. Gene transcription in the brains of chickens infected with H5N1 or velogenic neurotropic NDV have been analyzed using Gene Fishing™, which is an mRNA differential display technique. Several key factors involved in neural signal transduction, the cytoskeletal system, or protein folding during stress were found to be significantly regulated during infection by Highly Pathogenic Avian Influenza (HPAI) or NDV [28]. Gene expression profiling in the spleen during JS5/05 (genotype VIId NDV strain) and Herts/33 (genotype IV NDV strain) infection has also been performed. JS5/05 induced a more severe inflammatory response, which may be related to the pathogenicity of the virus [29]. 

Theoretically, one gene can be regulated by thousands of miRNAs, and one miRNA could have hundreds to thousands of targets [17,30]. The determination of a real interaction relationships between miRNAs and mRNAs is challenging using traditional methods, particularly in a very complex viral infection background. Big data analysis systems provide a useful tool for revealing the complex regulatory relationships between miRNA and their target(s). Inverse correlations between miRNAs and mRNAs can be identified using multi-omics joint analyses [31,32]. Although miRNA and mRNA transcription in cells and tissues after NDV infection has been previously analyzed, the correlation between miRNA and mRNA transcription has not been analyzed.

In this study, miRNAs and the gene expression profiles in visceral tissues from specific pathogen-free (SPF) chicken embryos infected with different virulent NDVs (F48E9 and La Sota) were investigated. A combined analysis of miRNA sequencing and the mRNA transcriptome could provide a more comprehensive and accurate understanding of gene alterations in chicken embryos infected by NDV. Our findings could provide new insight into the pathogenesis and immune mechanisms of NDV infection. 

## 2. Results

### 2.1. NDV Infection, Clinical Symptoms, and Sample Collection 

The SPF chicken embryos were inoculated with 10^4^ plaque-forming units (PFU) of NDV F48E9 or La Sota. NDV infection was confirmed by testing the hemagglutinin (HA) titers at 36 hpi when the embryos infected by F48E9 showed less vitality. The HA titers of F48E9 and La Sota in allantoic fluids were both 2^8^. No HA titer was detected in the control group, indicating no viral contamination during virus infection and harvest. In addition, the infected embryos, particularly those infected by F48E9, exhibited typical NDV-related infection symptoms, including reduced vitality and serious hemorrhage (Figure S1). No clinical signs were observed in the control chicken embryos during the experiment. The visceral tissues were collected from each group and subsequently used for small RNA (sRNA) and transcriptome sequencing.

### 2.2. Global miRNA Expression Pattern in Visceral Tissues from Chicken Embryos

**Figure 1:**
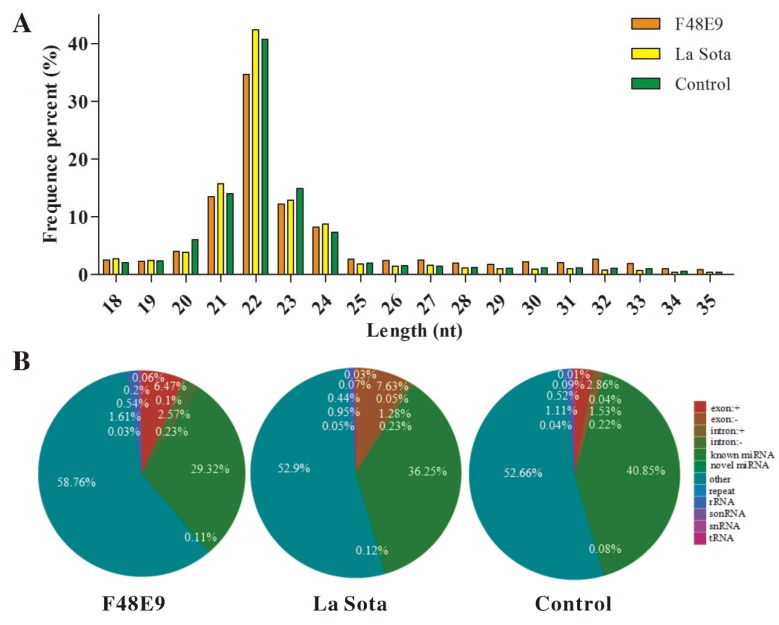

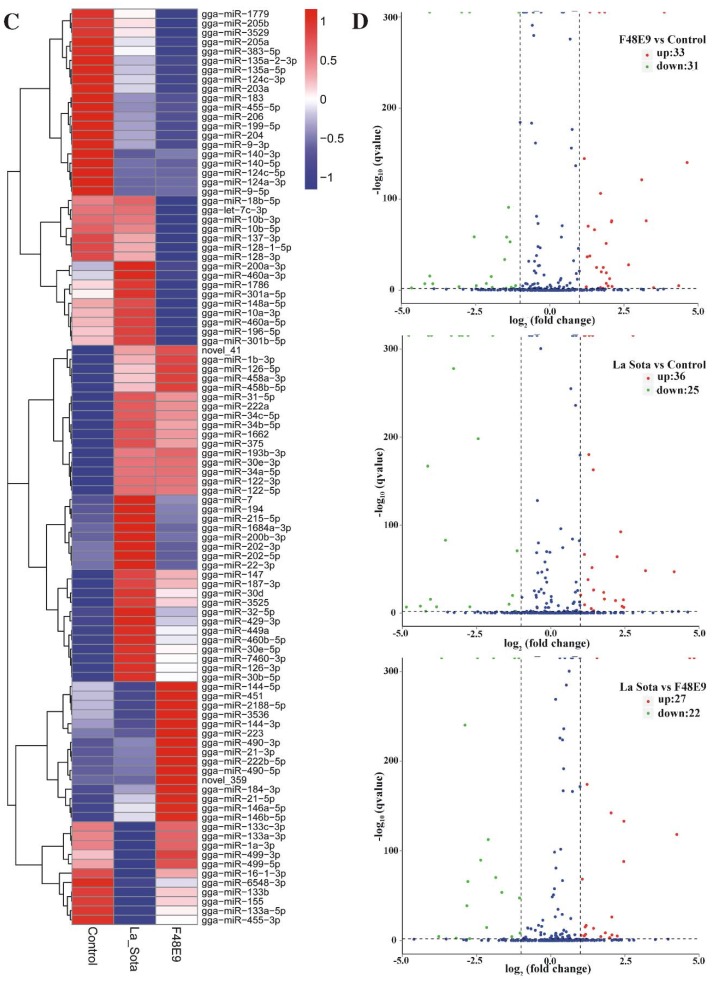

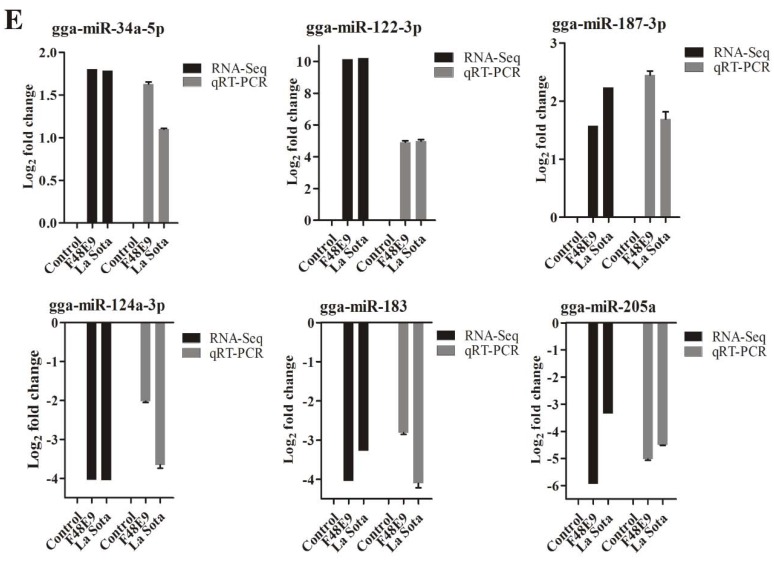
Different expression profiles of microRNAs in chicken embryos infected with F48E9 or La Sota. (**A**) Size distribution of sequenced small RNA-seq reads. (**B**) Pie charts of small RNA-seq showing the percentage of small RNA components in F48E9 or La Sota infected tissue and control tissue. (**C**) Heatmap of 98 differentially expressed miRNAs shared by F48E9, La Sota, and the control. (**D**) Scatter plots showing the upregulated and downregulated differentially expressed miRNAs between F48E9 and control, La Sota and control, and La Sota and F48E9. Red, green and blue dots are representative the number of upregulated, downregulated and unchanged genes, respectively. (**E**) Validation of RT-qPCR analysis of gga-miR-34a-5p, gga-miR-122-3p, gga-miR-187-3p, gga-miR-124a-3p, gga-miR-183, and gga-miR-205a in different infected and non-infected tissues.

In total, 11,382,301, 11,852,540, and 12,423,522 raw reads and 1,038,908, 777,804, and 803,645 unique reads were obtained from the libraries derived from SPF chicken embryonic tissues inoculated with F48E9, La Sota, or PBS, respectively. The raw sequences were filtered to obtain the clean reads, which were then mapped to the reference genome sequence of chickens. In total, 8,545,608 (85.86%), 9,206,624 (89.24%), and 1,0007,533 (88.28%) reads from F48E9, La Sota, and the control (C) group were successfully mapped to the *Gallus gallus* genome. Most clean reads were 21–24 nt in length, and the 22 nt sRNAs were the most abundant (Figure 1A). The numbers of miRNAs were 2,505,516 (29.32%), 3,337,395 (36.25%), and 4,087,620 (40.85%) in the F48E9, La Sota, and C groups, respectively (Figure 1B). Among these miRNAs, the numbers of mapped unique sRNA (known as miRNAs) and novel miRNAs were 5109 and 80, 5453 and 98, and 5934 and 120 in the F48E9, La Sota, and C groups, respectively.

The identification of differentially expressed miRNAs between the infected and uninfected groups was performed on the basis of a *q*-value threshold < 0.01 and |log2 (fold change)| > 1. By analyzing a volcano plot based on their expression, three differentially expressed clusters containing 98 miRNAs were identified (Figure 1C and Table S1). Accordingly, F48E9 infection was found to increase the expression of 33 miRNAs and decrease the expression of 31 miRNAs, while La Sota infection upregulated 36 and downregulated 25 miRNAs. Meanwhile, 56 miRNAs were differentially regulated by La Sota and F48E9, including 27 up- and 28 downregulated miRNAs (Figure 1D). This finding suggested that different miRNA regulation characteristics exist among NDV strains with different degrees of virulence.

To validate the data obtained from the RNA-Seq, six randomly selected miRNAs, including gga-miR-122-3p, gga-miR-187-3p, gga-miR-34a-5p, gga-miR-124a-3p, gga-miR-205a, and gga-miR-183, were examined using reverse transcription quantitative polymerase chain reaction (RT-qPCR). The expression levels of the miRNAs obtained by both methods (RNA-Seq and RT-qPCR) were comparable, indicating that the data obtained by RNA-Seq were reliable (Figure 1E).

### 2.3. Prediction and Functional Characterization of Potential Target mRNAs of Common miRNAs during NDV Infection

**Figure 2:**
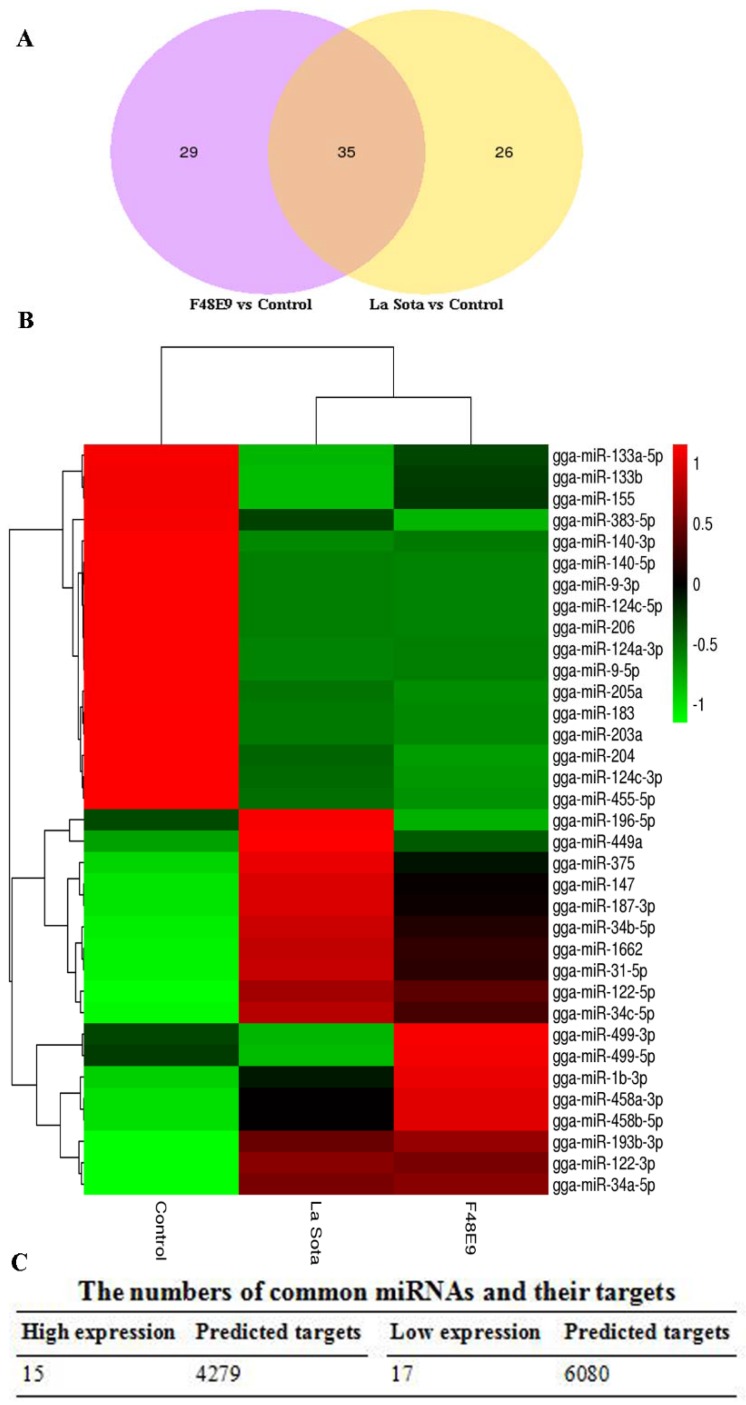

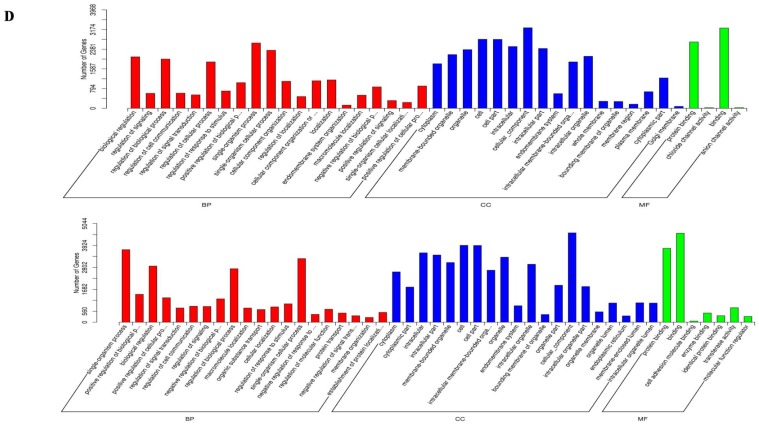

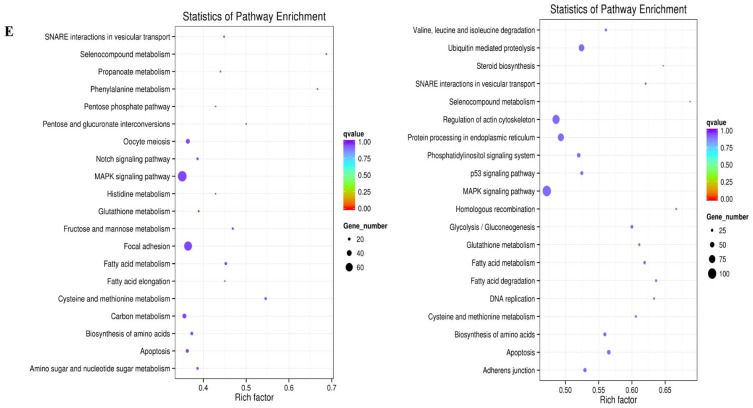
Common up- and downregulated miRNAs and their target genes in response to Newcastle disease virus (NDV) infection. (**A**) Venn diagram showing the number of common differentially expressed miRNAs in F48E9 and La Sota infections. Purple and yellow represent the differentially expressed miRNAs after infection with F48E9 and La Sota, respectively, and the common parts represent the same differentially expressed miRNAs when infected with F48E9 and La Sota. The numbers in each section indicate the numbers of differentially expressed miRNAs. (**B**) Heatmap of 35 common differentially expressed miRNAs in both F48E9 and La Sota infections. (**C**) Statistical table showing the number of common up- and downregulated miRNAs and the numbers of their predicted target mRNAs. (**D**) GO analysis of the predicted target genes with common up- and downregulated miRNAs in NDV infection. The top panel shows the GO enrichment analysis of each of the top 20 significant differences in the upregulated miRNAs, and the lower panel shows the GO enrichment analysis of each of the top 20 significant differences in the downregulated miRNAs. (**E**) KEGG analysis of the predicted target genes with common up- and downregulated miRNAs in NDV infection. The left panel shows the KEGG enrichment analysis of each of the top 20 significant differences in the upregulated miRNAs, and the right panel shows the KEGG enrichment analysis of each of the top 20 significant differences in the downregulated miRNAs.

A Venn diagram was generated to display the differentially expressed miRNAs between La Sota and F48E9 infection. As shown in Figure 2A, 35 miRNAs in the chicken embryos changed their expression levels during La Sota and F48E9 infections (common changes). In addition, 26 miRNAs altered their expression levels after La Sota infection (strain specific), and 29 miRNAs changed after the F48E9 infection. The expression levels of gga-miR-196-5p, gga-miR-499-3p, and gga-miR-499-5p were altered by both the La Sota and F48E9 infections; however, these miRNAs were differentially regulated by these two viruses with different virulence levels (Figure 2B). Except for the abovementioned three miRNAs, 15 and 17 miRNAs were up- or downregulated by both viruses (Figure 2B,C).

The targets of these miRNAs were predicted using the online software miRanda. The 15 upregulated miRNAs had 4279 targets, and the 17 downregulated miRNAs had 6080 targets (Figure 2C and Table S2). Then, Gene Ontology (GO) and Kyoto Encyclopedia of Genes and Genomes (KEGG) functional enrichments were performed to explore the distribution and potential biological functions of these 10359 candidate target genes. 

On the basis of the GO biological processes, the candidate targets may be involved in biological processes or be cellular components but are less likely to participate in molecular functions. Among these processes, the biological processes related to the targets of the upregulated miRNAs included cell death, apoptotic processes, protein transport, positive regulation of biological processes, and regulation of a cellular metabolic process (Figure 2D upper and Table S3). The unique functions of the candidate targets of the downregulated miRNAs were connected with signaling of metabolic processes, cellular localization, negative regulation of biological processes, and positive regulation of cellular processes (Figure 2D lower and Table S3).

The candidate targets involved in the most important biochemical, metabolic, and signal transduction pathways were identified using KEGG pathway mapping. The 20 most prominent KEGG pathways are exhibited in Figure 2E. Accordingly, the targets of these upregulated miRNAs may participate in numerous biological processes, such as SNARE interactions in vesicular transport, Notch signaling pathway, oocyte meiosis, MAPK signaling pathway, focal adhesion, apoptosis, etc. (Figure 2E left and Table S4). In addition, the targets of the downregulated miRNAs were involved in other types of pathways, including the p53 signaling pathway, ubiquitin mediated proteolysis, DNA replication, etc. (Figure 2E right and Table S4). 

### 2.4. Global mRNA Expression Pattern in Visceral Tissues from Chicken Embryos during NDV Infection

**Figure 3:**
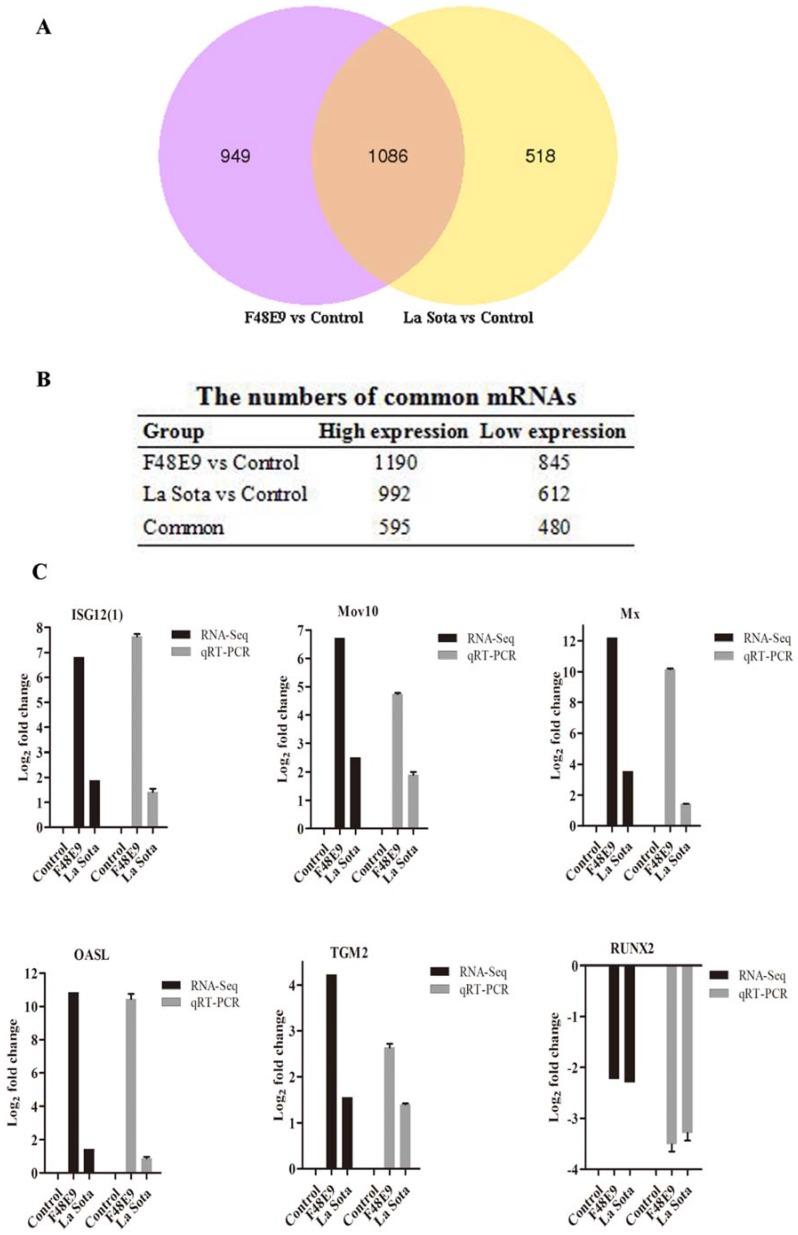

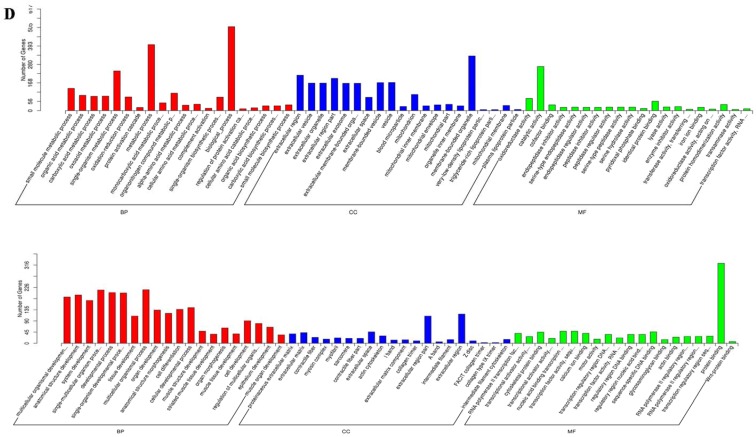

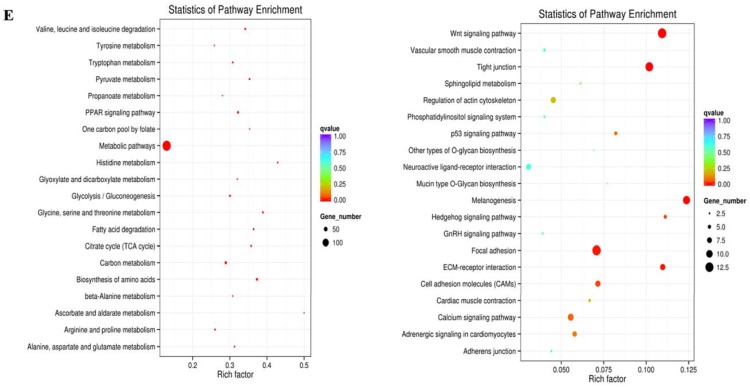
mRNA expression profiles in NDV-infected chicken embryonic tissues. (**A**) Venn diagram of RNA-Seq showing the number of common up- and downregulated mRNAs in F48E9 and La Sota infections. Purple and yellow represent the differentially expressed genes after infection with F48E9 and La Sota, respectively, and the common parts represent the same differentially expressed genes when infected with F48E9 and La Sota. The numbers in each section indicate the numbers of differentially expressed mRNAs. (**B**) Statistical table showing the number of common up- and downregulated mRNAs in response to NDV infection. (**C**) Validation of RT-qPCR analysis of ISG12(1), Mov10, Mx, OASL, TGM2, and RUNX2 in different infected and non-infected tissues. (**D**) GO analysis of the common up- and downregulated mRNAs in NDV infection. The top panel shows the GO enrichment analysis of each of the top 20 significant differences in the upregulated mRNAs, and the bottom panel shows the GO enrichment analysis of each of the top 20 significant differences in the downregulated mRNAs. (**E**) KEGG analysis of the common up- and downregulated mRNAs in NDV infection. The left panel shows the KEGG enrichment analysis of each of the top 20 significant differences in the upregulated mRNAs, and the right panel shows the KEGG enrichment analysis of each of the top 20 significant differences in the downregulated mRNAs.

To investigate the mRNA expression profiles during NDV (F48E9 or La Sota) infection, the gene transcripts were analyzed using RNA-Seq. Compared with those from the uninfected group, 2035 (1190 up- and 845 downregulated) and 1604 (992 up- and 612 downregulated) differentially expressed mRNAs were identified in the F48E9- and La Sota-infected samples, respectively (Figure 3A,B and Table S5). A Venn diagram was generated to illustrate the changes induced by NDV infection, and 1086 mRNAs were found to be altered by both F48E9 and La Sota infections (Figure 3A). Of these 1086 mRNAs, 1075 mRNAs showed the same tendency, including 595 upregulated and 480 downregulated (Figure 3B and Table S6), while other 11 genes (CSRP3, MYL3, MYL2, MYBPC3, MME, POPDC2, BVES, myosin, Gadd45, ENSGALG00000018386, and Novel00060) were oppositely regulated by the F48E9 and La Sota infections.

To verify the data obtained from the deep sequencing, six randomly selected mRNAs, including Mov10, TGM2, Mx, OASL, RUNX2, and ISG12(1), were detected using RT-qPCR. The expression tendencies of these mRNAs detected by RT-qPCR were similar to those obtained by high-throughput sequencing (Figure 3C), indicating that the RNA-Seq data were reliable. A GO-based analysis and KEGG mapping were performed to explore the biological functions of the 1075 mRNAs regulated by both the F48E9 and La Sota infections (Figure 3D,E). According to the GO analysis, the upregulated genes played a role in the regulation of innate immunity, inflammatory response, response to viruses, apoptosis, cell activation, and biological processes, including cell-type specific apoptotic processes, sequence-specific DNA binding, and transcription factor activity (Figure 3D upper and Table S7). Similarly, the downregulated genes were involved in the innate immune response, humoral immune response, inflammatory response, cell death, apoptosis process, and biological processes of the host (Figure 3D lower and Table S7). According to the KEGG mapping, these genes are involved in the RIG-I-like receptor signaling pathway, Toll-like receptor signaling pathway, NOD-like receptor signaling pathway, response to influenza A, apoptosis, Jak–STAT signaling pathway, cell cycle, endocytosis, MAPK signaling pathway, ECM–receptor interactions, Wnt signaling pathway, and cytokine–cytokine receptor interactions (Figure 3E and Table S8).

### 2.5. Conjoint Analysis of Small RNA-Seq and mRNA-Seq

**Figure 4:**
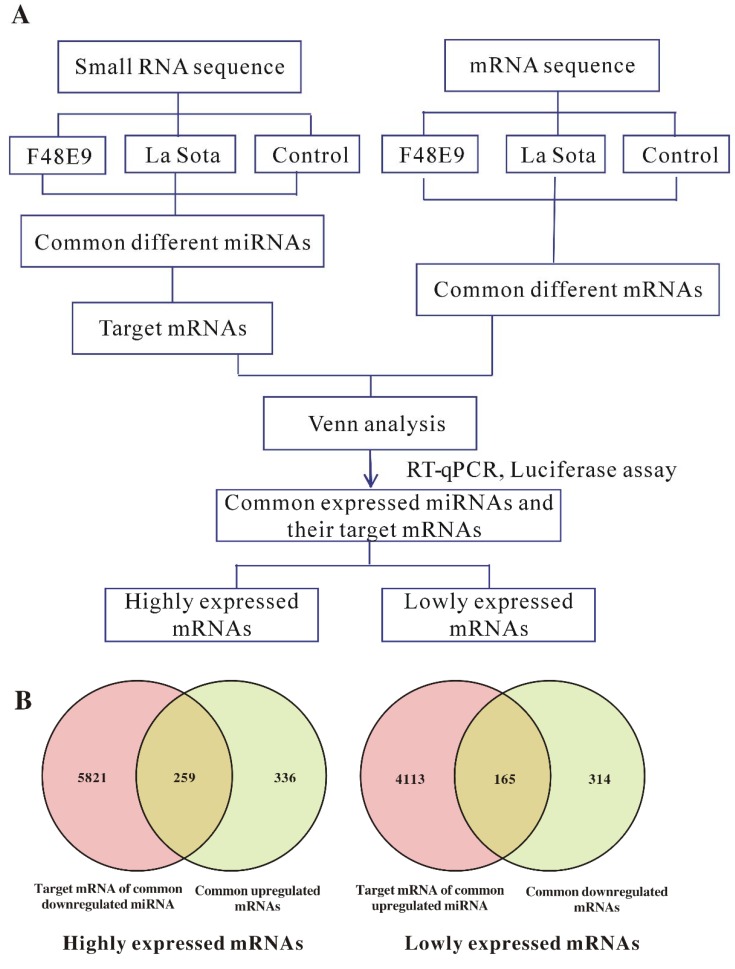

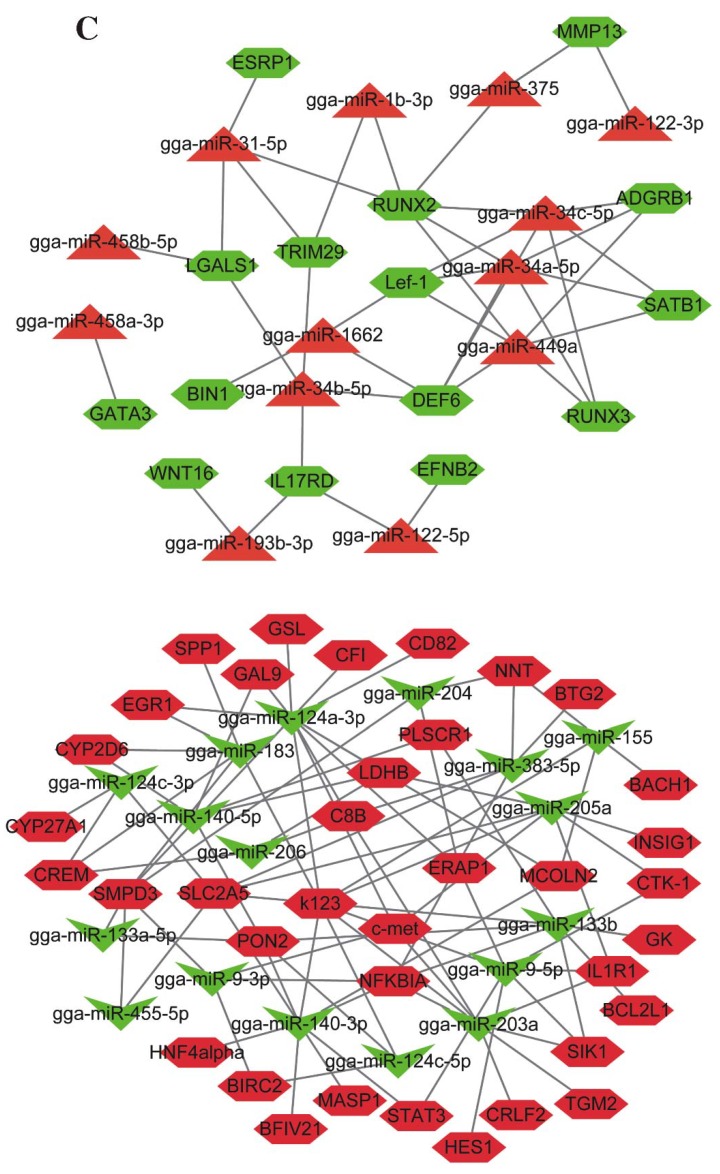
Conjoint analysis of small RNA-seq and RNA-seq in chicken embryos infected with NDV. (**A**) Flow diagram of the conjoint analysis of small RNA-seq and RNA-seq. (**B**) Venn analysis of the predicted miRNA target mRNAs and the differentially expressed mRNAs with the same expression trend in both the F48E9 and La Sota infections. The left panel shows the common highly expressed mRNAs, and the right panel shows the common lowly expressed mRNAs. (**C**) miRNA–mRNA interaction networks in NDV-infected chicken embryonic tissues. The top panel shows the miRNA–mRNA network of the upregulated miRNAs and downregulated mRNAs, and the bottom panel shows the miRNA–mRNA network of the downregulated miRNAs and upregulated mRNAs.

A conjoint analysis of small RNA-Seq and mRNA-Seq was performed according to the strategy shown in Figure 4A. In total, 165 downregulated and 259 upregulated mRNAs were identified on the basis of a Venn map of the miRNA targets and common differentially expressed genes (DEGs) (Figure 4B and Table S9). These common targets (Tables S10 and S11) were conjointly analyzed in terms of enriched KEGG pathways and functionally enriched GO terms. The highly expressed target genes during both the F48E9 and La Sota infections were enriched in typical GO terms and pathways related to immune response or cell regulation, such as the RIG-I-like receptor signaling pathway, Jak–STAT signaling pathway, apoptosis, RNA degradation, PPAR signaling pathway, etc. (Tables S10 and S11). However, the lowly expressed target genes during NDV infection were mainly involved in cell differentiation and metabolism, such as in the p53 signaling pathway, Wnt signaling pathway, Notch signaling pathway, mTOR signaling pathway, etc. (Tables S10 and S11). Interaction networks of the common up- and downregulated miRNAs and the target mRNAs were constructed (Table S12). The miRNA–mRNA pairs involved in immunity and inflammatory responses were selected and are shown in Figure 4C; in total, 40 upregulated miRNA and downregulated mRNA pairs (Figure 4C upper) and 90 downregulated miRNA and upregulated mRNA pairs (Figure 4C lower) were identified. Interestingly, several novel miRNA–mRNA pairs were discovered in the correlation analysis, including miR-203a-TGM2, miR-122-5p-IL-17RD, miR-205a-IL1R1, miR-9-5p-STAT3, miR-383-5p-NFKBIA, miR-124a-3p-CD82, etc.

### 2.6. Validation of the miRNA–mRNA Interactions and Example of a Related Biological Function in NDV Replication

**Figure 5:**
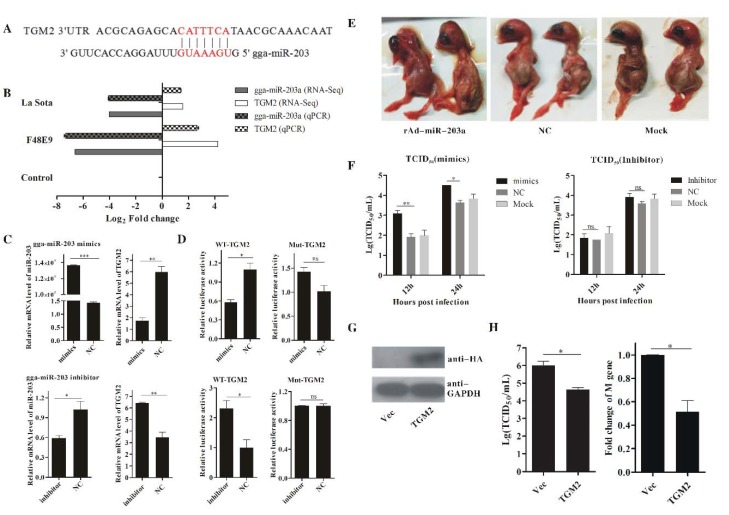
Regulation of transglutaminase 2 (TGM2) by gga-miR-203a and NDV replication. (**A**) Predicted binding sites of gga-miR-203a at the 3′UTR region in the TGM2 sequence. The red labeled sequence represents the complementary region between gga-miR-203a and the 3′ UTR of TGM2. (**B**) RNA-Seq and RT-qPCR detected expression changes of gga-miR-203a and TGM2 during NDV infection. (**C**) RT-qPCR detected expression regulation of TGM2 by gga-miR-203a. (**D**) A luciferase activity assay detected expression regulation of TGM2 by gga-miR-203a. WT is representative of wild-type TGM2, and Mut is representative of TGM2 with mutations in the region having complementarity with the miRNA. (**E**) Clinical changes in chicken embryos submitted to rAd-miR-203, negative control (NC), or PBS infections; NC indicates rAd-miR-NC and Mock indicates treatment consisting of an injection with the same volume of PBS. (**F**) TCID_50_ (50% tissue culture infection doses) detection of the viral titers of NDV in DF-1 cells treated with gga-miR-203a mimics or inhibitors. NC is the negative control, and Mock is representative of cells transfected only with the transfection reagents. (**G**) Western blot analysis detecting the expression of TGM2; Vec is representative of the empty vector. (**H**) RT-qPCR and TCID_50_ detection of the viral titers of NDV in DF-1 cells with TGM2 overexpression; Vec is representative of the empty vector. The results are presented as the means ± SEM (*n* = 3). The statistical analyses were performed in GraphPad Prism using unpaired 2-tailed *t*-tests: * *p* < 0.05, ** *p* < 0.01, *** *p* < 0.001, ns. indicates no significant difference.

To validate the correlations between the miRNAs and mRNAs, the predicated bond between gga-miR-203a and transglutaminase 2 (TGM2) (Figure 5A) was further studied. Both the RNA-Seq and RT-qPCR results indicated that gga-miR-203a and its target gene TGM2 had an inverse expression tendency in both F48E9 and La Sota infections, with 4.08~10.85 log_2-fold_ changes (Figure 5B). The expression of TGM2 in DF-1 cells treated with gga-miR-203a mimics or inhibitors was quantified using RT-qPCR. Compared with the negative control, the overexpression of or interference with gga-miR-203a could significantly suppress or increase the expression of TGM2 (Figure 5C). The luciferase assay confirmed the inverse relationship between gga-miR-203a and TGM2 (Figure 5D). 

To investigate the biological function of gga-miR-203a in NDV replication, NDV proliferation in both DF-1 cells and chicken embryos treated with gga-miR-203a mimics or rAd-miR-203 was detected. As shown in Figure 5E,F, the excessive expression of gga-miR-203a significantly accelerated embryonic death and NDV replication. In contrast, the overexpression of TGM2 obviously inhibited NDV replication either at the mRNA level or at the virion level (Figure 5G,H). Thus, gga-miR-203a regulated the expression of TGM2, which plays a negative role in NDV infection.

## 3. Discussion

miRNAs play an important role in the regulation of the pathogenetic processes of disease and the innate and adaptive immunity of the host [15,18,33]; however, their roles in the regulation of the responses to NDV infection in chicken embryos are unclear. Recently, deep sequencing with a low operation cost and high-throughput analysis has become a powerful tool in identifying the complex correlation between miRNAs and their potential targets during viral infection [11,12,13,34]. NDV is among the most infectious causative agents of viral diseases in birds and causes substantial losses to the poultry industry [3]. Chicken embryos are usually used to isolate and amplify NDVs and are also applied in research studies in the fields of virology, neurology, development, oncology, vaccine development, model animals, etc. [35,36]. To reveal the interaction between chicken embryos and NDV, the transcription patterns of miRNAs and mRNAs were obtained using deep sequencing. 

To the best of our knowledge, this study is the first to report an analysis of miRNA variation in visceral tissues from chicken embryos during NDV infection. In the present study, 64 (33 up- and 31 downregulated) and 61 (36 up- and 25 downregulated) differentially expressed miRNAs in response to NDV (F48E9 and La Sota) infection were identified. We revealed that 49 miRNAs (27 upregulated and 22 downregulated) were differentially regulated by the vetogenic and lentogenic NDV strains. Meanwhile, 32 miRNAs shared the same tendency in response to the F48E9 and La Sota infections. Among these miRNAs, several miRNAs, such as miR-9, miR-203a, miR-375, miR-122-5p, miR-147, etc., have been reported to play roles in the regulation of host immune responses. As previously reported, miR-9 inhibits type I IFN production by targeting interferon regulatory factor 2 (IRF2) to negatively regulate the host antiviral immune response [20]. miR-203 inhibits skin immune responses by targeting TNFα and IL24 [37]. miR-375 regulates *Helicobacter pylori*-induced inflammation by targeting the Jak2–STAT3 pathway, which subsequently promotes neoplastic transformation by affecting the expression of TWIST1 and BCL-2 [38]. In addition to gga-miR-499a-3p, gga-miR-499a-5p and gga-miR-169, and 4279 and 6080 target genes were predicted for the 15 upregulated and 17 downregulated miRNAs, respectively. The analysis of the candidate target genes that were regulated by the differentially expressed miRNAs based on GO or KEGG pathway enrichments revealed that the target genes were involved in many signaling pathways, including metabolism, development, immunity, apoptosis, etc. Thus, a variety of miRNAs were differentially expressed during NDV infection, and different regulatory mechanisms of miRNA expression may exist between velogenic and lentogenic NDV infection. 

The data of the viral gene transcription and host mRNA transcriptome analyses have been reported in another paper. Here, our analysis focused on the genes regulated by vetogenic and lentogenic NDV in a similar direction. Thus, 595 upregulated and 480 downregulated host genes had a common response to the F48E9 and La Sota infections. The biological analysis indicated that the DEGs were mainly enriched in the following pathways: cell differentiation, material metabolism, immunity, apoptosis, etc. The expression of osteopontin (OPN), which is related to host immunity, was upregulated in response to NDV infection. OPN selectively couples with TLR9 signaling to induce transcription factor IRF-7-dependent IFN-α expression, which plays an essential role in antigen cross-presentation in vitro and anti-herpes simplex virus 1-associated IFN-α response in vivo in plasmacytoid dendritic cells (pDCs) [39]. Signal transducer and activator of transcription 5 (STAT5), which is involved in the regulation of vital cellular functions, including proliferation, differentiation, survival, and immunity, by regulating specific cytokine members of the IL-2 family [40,41], also showed a high level of expression during NDV infection. In addition, the high expression of inhibitor of apoptosis protein (IAP), which is a vital regulator of cell death and survival pathways, was also observed during NDV infection. IAP modulates innate and adaptive immunity by affecting signal transduction pathways, cytokine production, and cell survival [42,43]. Although some finding is similar to those reported in previous studies performing transcriptome analyses of cells or chicken tissues infected with NDV, in this study, the expression of many genes related to embryonic development was changed in response to NDV infection. These genes, including nuclear receptor subfamily 5 group A member 2 (NR5A2), HNF1 homeobox B (HNF1B), forkhead box F1 (FOXF1), GATA binding protein 4 (GATA4), vascular endothelial growth factor A (VEGFA), prospero homeobox 1 (PROX1), etc., are involved in embryonic processes or in their regulation. Thus, embryonic development and tissue differentiation are strongly influenced by NDV infection, leading to deformity or embryonic death. 

To increase the reliability of the results, the correlations between the obtained small RNA and mRNA data were analyzed. The inverse correlations, according to the function of the miRNAs on the mRNAs [32], were our focal point. Most miRNAs were inversely correlated with more than one mRNA target, and one mRNA was also targeted by several miRNAs. Based on the inverse correlation analysis, 1069 miRNA–mRNA pairs (431 mRNAs connected to 15 upregulated miRNAs and 638 mRNAs connected to 17 downregulated miRNA) were identified during NDV infection. The functional annotation analysis revealed that these potential targets were mainly involved in metabolism and development, and a few genes played roles in the regulation of inflammatory responses, apoptosis, cytokine expression, and immune responses. In total, 130 miRNA–mRNA pairs were involved in immune or inflammatory responses (Figure 4C). Interleukin-17 receptor D (IL-17RD) and its partner miR-223-3p may play a role in the regulation of the immune response against NDV infection. IL-17RD was found to negatively regulate Toll-like receptor (TLR)-induced immune responses by targeting TIR adaptor protein, which sequentially inhibited the downstream signaling of TLR [44]. Furthermore, the heteromer of IL-17RD and TNF receptor 2 (TNFR2) has been reported to play a role in the activation of NF-κB signaling [45], and a relationship between IL-17RD and miR-223-3p has been identified in previous studies [46]. In the current study, gga-miR122-5p, gga-miR-193b-3p, gga-miR-34b-5p, and miR-223-3p were negatively correlated with IL-17RD during NDV infection, indicating that IL-17RD may play an important role in NDV infection. In addition, we found that gga-miR-140-3p and gga-miR-9-5p were inversely correlated with signal transducer and activator of transcription 3 (STAT3), which plays a key role in regulating host immune responses in several viral diseases [47,48], and gga-miR-9-5p, gga-miR-203a, and gga-miR-205a likely downregulate IL-1R1 expression because their expression was negatively correlated with IL-1R1 expression in our analysis. IL-1 signaling plays an important role in the induction of the inflammation response and early activation of host innate immune responses following virus infection. Viral microRNA derived from Epstein–Barr virus (EBV) blocked IL-1 signaling by targeting IL-1 receptor 1 and, consequently, altered the cytokine expression levels during EBV infection [49]. These data strongly support the hypothesis that miRNAs play important roles in the regulation of immune responses against NDV in chicken embryos.

The expression of toll-like receptor 4 (TLR4), tumor necrosis factor-α (TNF-α), and interleukin-12 (IL-12) decreased in response to treatment with miR-203 mimics in dendritic cells. IL-12 expression increased in cells treated with miR-203 inhibitors [50]. In our study, the overexpression of gga-miR-203a effectively increased NDV replication both in vivo and in vitro. The potential target of gga-miR-203a, i.e., transglutaminase 2 (TGM2), plays a vital role in dendritic cell activation, B cell differentiation, CD8+ T cell generation, inflammation, etc. [51,52,53], and its expression was inversely correlated with the expression of gga-miR-203a in our study. Therefore, the relationship between gga-miR-203a and TGM2 was further validated using RT-qPCR and luciferase assays. According to their correlation, gga-miR-203a and TGM2 also play opposing roles in NDV replication. TGM2 activates the NF-κB pathway to generate inflammatory cytokines and chemokines that degrade IκB or activate latent transforming growth factor-β (TGF-β) [52,54]. Therefore, gga-miR-203a may suppress TGM2 to deactivate the NF-κB signaling pathway and consequently enhance NDV replication. These results partially confirm the correlation between the obtained miRNA–mRNA pairs; notably, the specific regulatory relationships of the other miRNA–mRNA pairs require further validation. The GO enrichment of the target genes of gga-miR-203a showed that TGM2 plays a significant role in extracellular regions, vesicles, and exosomes, and we hypothesize that TGM2 may be involved in miRNA exocytosis- or membrane fusion-mediated IFN production, affecting viral replication [55,56]. Similar reasoning could apply to many other miRNAs and their targets, and these findings can enlighten a series of interesting studies in related fields.

## 4. Materials and Methods

### 4.1. Ethics Statement

The experimental use of chicken embryos in this study was operated on strictly according to the Committee for the Ethics of Animal Care and Experiments in Northwest A&F University (Approval number: 2015ZX08008016-016), which was approval at 8 September in 2015.

### 4.2. Virus, Cells, and Chicken Embryos

The velogenic NDV strain, F48E9, and lentogenic NDV strain, rLa Sota-GFP (La Sota) were maintained in our laboratory. The viruses were propagated in 9- to 11-day-old SFP chicken embryos, titrated with HA assays, and stored at −80 °C. All incubations and reactions were performed in 5% CO_2_ and at 37 °C in flat-bottom plates following the manufacturer’s instructions. DF-1 and HEK 293T cells were maintained and cultured in Dulbecco’s modified Eagle’s medium (DMEM) with 10% fetal bovine serum (FBS, Gibco, New York, NY, USA). The 10-day-old SPF chicken embryos were purchased from Jinan SAIS Poultry Company (Shangdong, China) and incubated at 37 °C. 

### 4.3. NDV Infection, Sample Collection, and Preparation

F48E9 or La Sota were independently injected into the allantoic cavity of 10-day-old chicken embryos at a dosage of 10^4^ PFU. The same volume of PBS was injected as a negative control. Visceral tissues were collected from each group at 36 hpi and then sent to Novogene for sequencing (Wuhan, China).

Total RNA was extracted from each pool of visceral tissues using an RNAiso Plus kit (Takara, Dalian, China) according to the manufacturer’s instructions. The collected RNA was treated with PQI DNase (Promega, Fitchburg, MI, USA) to eliminate DNA contamination. The RNA concentration and quality (260/280 ratio) were assessed using a NanoDrop spectrophotometer 2000c (NanoDrop Technologies, Wilmington, DE, USA). The RNA degradation was monitored on 1% agarose gels, and the integrity of the RNA samples was also assessed using an RNA Nano 6000 Assay Kit on a Bioanalyzer 2100 system (Agilent Technologies, Santa Clara, CA, USA) according to the manufacturer’s instructions.

### 4.4. Library Construction and Deep Sequencing

The RNA extraction, library preparation, and sequencing analyses were performed by the Novogene Company (Beijing, China). In brief, 3 μg of each pooled total RNA was used to create small-RNA libraries. Small RNA was generated using a NEBNext Multiplex Small RNA Library Prep Kit (NEB, Boston, MA, USA) following the manufacturer’s recommendations. First, 3′ or 5′ adapters were ligated to the 3′ or 5′ ends of all sRNA using T4 RNA ligase. Then, the libraries were used as templates for the synthesis of the first-strand cDNA using M-MuLV reverse transcriptase. Finally, PCR amplification was conducted, and the products with a length in the range of 18–35 nt were purified using polyacrylamide gel electrophoresis. The cDNA library quality was assessed using a Bioanalyzer 2100 system (Agilent Technologies, Santa Clara, CA, USA) and adjusted to a final concentration of 1 ng/µL. Then, the libraries were sequenced on an Illumina HiSeq^TM^ 2500 platform.

Three cDNA libraries were also generated using an UltraTM RNA Library Prep Kit (NEB, Boston, MA, USA) following the manufacturer’s recommendations. cDNA fragments with a length of 150–200 bp were purified using an AMPure XP system (Beckman Coulter, Beverly, MA, USA). The purified DNA fragments were enriched and purified (AMPure XP system), and the library quality was assessed using an Agilent Bioanalyzer 2100 system. After performing the clustering of the index-coded samples using a cBot Cluster Generation System and a TruSeq PE Cluster Kit (Illumina, San Diego, CA, USA), the sequencing runs were performed on an Illumina HiSeq platform. 

### 4.5. Screening for Differentially Expressed miRNAs and mRNAs

The raw data from both the mRNA and miRNA sequencing were submitted to the GEO of NCBI with accession numbers GSM3042966, GSM3042967, and GSM3042968 for the mRNA data and GSM3042969, GSM3042970, and GSM3042971 for the miRNA data of the F48E9, La Sota, and control groups, respectively.

Before submitting to the downstream analytical pipelines, the raw reads underwent quality trimming, and, then, the Q20, Q30, and GC contents were calculated. Clean reads with Q Score > 30 were selected and aligned with the corresponding paired reads. Finally, sequences with lengths of 18–35 nt were used for the differential expression analysis. The selected clean reads from each sample were aligned and mapped to the *Gallus gallus* reference genome (Available online: ftp://ftp.ensembl.org/pub/release-81/fasta/gallus_gallus/dna/) using Bowtie. Detailed information regarding the mapped sRNAs, including the expression abundance, length, and sequence of the known and novel miRNAs, was obtained by analyzing sequences using miRBase. To detect the differentially expressed miRNAs, corrected *p*-value (*q*-value) < 0.01 and |log2 (foldchange)| > 1 were set as the threshold parameters for the significant miRNAs. 

The host gene transcription was analyzed by mapping the clean reads to the chicken reference genome (*Gallus gallus* reference genome Galgal 4.81) using TopHat2. To detect the differentially expressed genes (DEGs), the number of clean reads assigned to a gene was counted using HTSeq v0.6.1 [57] and then normalized to the values of fragments per kilobase of exon per million fragments mapped (FPKM) [58]. The differential expression levels among the groups were analyzed using the DEGSeq R package (1.20.0) [59]. Corrected *p*-value (*q*-value) < 0.005 and |log2 (foldchange)| > 1 were set as the threshold parameters for the significant DEGs.

### 4.6. Functional Analysis

miRanda 3.3a was used for the prediction of the targets of the differentially expressed miRNAs. A GO functional enrichment analysis and KEGG pathway enrichment analysis were performed for the prediction of the functions of these miRNAs or their targets using the Goseq R package and KOBAS 2.0 software (Available online: http://kobas.cbi.pku.edu.cn/home.do) [60]. 

### 4.7. Correlation Analysis of miRNAs and mRNAs 

To build the miRNA–mRNA interaction network, a negative correlation analysis of miRNAs and mRNAs was performed. The biological functions of the selected genes were analyzed using GO and KEGG pathway enrichment, and Cytoscape v2.8.3 software (Available online: http://www.cytoscape.org/) was used to construct the miRNA–mRNA interaction network. 

### 4.8. Real Time qPCR (RT-qPCR)

Relative quantification methods were used to analyze the expression of the target miRNAs and mRNAs. The primers used in this study are listed in Table 1. Real-time qPCR was conducted using miRcute miRNA qPCR Detection Kit (TIANGEN, Beijing, China) for miRNAs or 2× RealStar green power mixture (GenStar, Beijing, China) for mRNAs, according to the manufacturer’ instructions. The relative expression levels of the target genes were calculated using 2^−ΔΔ*C*T^ as previously described [61], and 28S was used to normalize the fold changes in expression. The experiments were repeated independently at least three times.

### 4.9. Gene Clone and Vector Construction

The gga-miR-203a mimics, inhibitor, and negative control (NC) were synthesized by GenePharma (Shanghai, China). The 3′UTR of TGM2 and its mutation were cloned into psiCHECK2 and named psiCHECK2-TGM2 (wild type, WT-TGM2) and psiCHECK2-TGM2-mut (Mut-TGM2). The target gene chTGM2 was cloned into pCAGGS with an HA tag and named pCAGGS-HA-chTGM2 (TGM2). All primers are listed in Table 1, and the recombinant plasmids, including WT-TGM2, Mut-TGM2, and TGM2, were confirmed by DNA sequencing. 

### 4.10. Cell Transfection and Luciferase Assay

The cell transfection was performed using Lipofectamine 2000 transfection reagent (Invitrogen; Thermo Fisher Scientific, Inc., CA, USA) according to the manufacturer’s instructions. In brief, 2 µL/well of transfection reagent was diluted in 50 µL Opti-MEM without FBS for 5 min and then mixed with additional 50 µL of Opti-MEM containing the target plasmids, which was prepared 20 min before. Finally, the mixture was added to the cell culture medium, and, then, the cells were cultured in 5% CO_2_ and at 37 °C for further study.

The luciferase reporter assay was performed as previously described with slight modifications [64]. Briefly, HEK-293T cells were co-transfected with 500 ng of WT-TGM2/Mut-TGM2/empty vector (Vec) plasmid and 500 ng gga-miR-203a of mimics, inhibitor, or miR-NC using Lipofectamine 2000 transfection reagent (Invitrogen), as described above. The cells were lysed 24 h after transfection, and the luciferase activity in the total cell lysates was measured using a dual-luciferase reporter assay system (Beyotime, Shanghai, China), according to the manufacturer’s instructions. The experiments were repeated independently at least three times.

### 4.11. Virus Titration

Tissue culture infection doses (TCID_50_) were used to quantify the viral titers. Briefly, DF-1 cells were seeded into 96-well plates 24 h before the virus infection. Ten-fold serial dilutions were used in the titrations, and each dilution had three replicates. Five days post-infection, the TCID_50_ was calculated using the Reed–Muench method [65]. The experiments were repeated independently at least three times.

### 4.12. rAd-miR-203a Construction, Packaging, and Infection

The recombinant human defective type 5 adenovirus expressing miR-203a or an empty wild type adenovirus control, designated rAd-miR-203a and rAd-miR-NC, respectively, were constructed and packaged by the company supplying the biological materials (Beijing, China). We perform our experiments by using the mesogenic NDV strain Shaanxi10, which was isolated by us and stored in our laboratory. First, 10^6^ TCID_50_/egg of each rAd-miRNAs or an equal amount of PBS was injected into the allantois cavity of five chicken embryos, and, after 24 h, 100 TCID_50_/egg of the Shaanxi10 NDV strain were inoculated into the eggs using the same methods. After three days of incubation at 37 °C, the chicken embryos were collected and observed. The lesions caused by the NDV infection were recorded. The experiments were repeated independently at three times.

### 4.13. Western Blotting

Western blotting was performed to analyze the overexpression of chTGM2 in DF-1 cells. After 48 h of transfection, the cells were washed twice with PBS and lysed for 10 min in RIPA buffer with the protease inhibitor PMSF on ice. The proteins were separated by 10% SDS-PAGE, electro blotted onto polyvinylidene difluoride (PVDF) membranes (Millipore, Billerica, MA, USA), and subsequently reacted with a mouse anti-HA monoclonal antibody (1:3000, CoWin Biosciences, Beijing, China) and a mouse anti-GAPDH monoclonal antibody (1:3000, CoWin Biosciences, China) for 2 h at room temperature (RT) to detect TGM2 and GAPDH, respectively. HRP-conjugated goat anti-mouse IgG (1:5000 dilutions, incubated for 1 h at RT, Sungene Biotech, Tianjing, China) served as a secondary antibody. The immunostained proteins were visualized using an ECL peroxidase substrate (Millipore) detection reagent system. The experiments were repeated independently at three times.

### 4.14. Statistical Analysis

All data are expressed as the mean ± standard error. To identify significant differences, comparisons were performed using one-way analysis of variance for viral load and Student’s *t*-tests for mRNA levels using GraphPad Prism 5 (GraphPad Software, San Diego, CA, USA). The results were considered statistically significant at *p*-values less than 0.05.

## 5. Conclusions

In this study, on the basis of a high-throughput sequencing of small RNAs and mRNAs, differentially expressed miRNAs and mRNAs in response to velogenic and lentogenic NDV infections were identified, and we performed an inverse correlation analysis of the miRNAs and mRNAs. Among the 1069 miRNA–mRNA pairs, 130 miRNA–mRNA pairs were involved in the regulation of immunity or inflammatory responses. The correlation between miR-203a and its target TGM2 was validated using a dual-luciferase reporter system and an RT-qPCR assay. To date, this study is the first to report information regarding small RNAs and mRNAs in chicken embryos infected with NDV, which may play a vital role in the prevention of NDV infection and enhance our understanding of the interaction between the host and the NDV.

## Figures and Tables

**Table 1:** Primers used in this study.

Primers	Sequences (5′-3′)	Methods	References
Mx	F: AAGCCTGAGCATGAGCAGAA	Real-time qPCR	[62]
	R: TCTCAGGCTGTCAACAAGATCAA		
OASL	F: AGATGTTGAAGCCGAAGTACCC	Real-time qPCR	[62]
	R: CTGAAGTCCTCCCTGCCTGT		
MOV10	F: CCAGGACTGACATAAGAACACC	Real-time qPCR	
	R: TTCCCACGGATGAACTCG		
TGM2	F: CACGACACCAACGGCAACC	Real-time qPCR	
	R: GACCTCCGCAAAGACGAA		
RUNX2	F: ACTTTGACAATAACTGTCCT	Real-time qPCR	[63]
	R: GACACCTACTCTCATACTGG		
ISG12(1)	F: GGGTTCCATAGCAGCCAAG	Real-time qPCR	
	R: CAACGAAAGAGAGCCCCGC		
gga-miR-122-3p	F: AACGCCATTATCACACTAAATA	Real-time qPCR	
gga-miR-187-3p	F: TCGTGTCTTGTGTTGCAGCC	Real-time qPCR	
gga-miR-34a-5p	F: TGGCAGTGTCTTAGCTGGTTGTT	Real-time qPCR	
gga-miR-124a-3p	F: TTAAGGCACGCGGTGAATGCCA	Real-time qPCR	
gga-miR-183	F: TATGGCACTGGTAGAATTCACTG	Real-time qPCR	
gga-miR-205a	F: TCCTTCATTCCACCGGAGTCTG	Real-time qPCR	
28S	F: GGTATGGGCCCGACGCT	Real-time qPCR	
	R: CCGATGCCGACGCTCAT		
gga-miR-203a-RT	CTCAACTGGTGTCGTGGAGTCGGCAATTCAGTTGAGCAAGTGGT	Real-time qPCR	
gga-miR-203a-qF	ACACTCCAGCTGGGGTGAAATGTTTAGGAC		
URP (unified reverse primer)	TGGTGTCGTGGAGTCG		
Luc-TGM2	F: CCGCTCGAGGGGCCCCCCGAGCCCCCACCCT	Luciferase	
	R: TTGCGGCCGCTGGCTTTAAAAGTGAAAAATAGAGGT		
Mut-TGM2	F: TCGAGCCCCTGGGACGCAGAGCAGTAAAGTTAACGCAAACAATAGAAAGACAATCGC	Luciferase	
	R: GGCCGCGATTGTCTTTCTATTGTTTGCGTTAACTTTACTGCTCTGCGTCCCAGGGGC		
pCAGGS-HA-TGM2	F: GGAATTCATGGGTGGACCGGGACCGGACGG	Gene clone	
	R: CGGCTAGCTCAAGCGTAGTCTGGGACGTCGTATGGGTACTTGGGCAGGGGTGCGATG		

## Supplementary Materials

The following are available online at http://www.mdpi.com/1422-0067/19/5/1291/s1.

## Abbreviations

NDV	Newcastle disease virus
miRNA	microRNA
3′UTR	3-untranslated region
AIV	Avian influenza virus
IBDV	Infectious Bursal disease virus
MDV	Marek’s disease virus
RIG-I	Retinoic acid inducible-gene I
HCV	Hepatitis C virus
CEFs	Chicken embryonic fibroblasts
SPF	Specific pathogen-free
HA	Hemagglutinin
GO	Gene Ontology
KEGG	Kyoto Encyclopedia of Genes and Genomes
MAPK	Mitogen-activated protein kinase
CSRP3	Cysteine- and glycine-rich protein 3
MYL2	Myosin light chain 2
MYBPC3	Myosin binding protein C, cardiac
MME	Membrane metalloendopeptidase
POPDC2	Popeye domain containing 2
BVES	Blood vessel epicardial substance
Gadd45	Growth arrest and DNA damage-inducible 45
Mov10	Mov10 RISC complex RNA helicase
Mx	Interferon-induced GTP-binding protein Mx
OASL	2′-5′-oligoadenylate synthetase like
RUNX2	Runt-related transcription factor 2
ISG12(1)	Interferon alpha inducible protein 27 like 1 (IFI27L1)
RT-qPCR	Real-time quantitative polymerase chain reaction
IRF2	Interferon regulatory factor 2
TWIST1	Twist family bHLH transcription factor 1
BCL-2	B cell leukemia/lymphoma 2
DEGs	Differentially expressed genes
OPN	Osteopontin
TLR9	Toll-like receptor 9
STAT5	Signal transducer and activator of transcription 5
IAP	Inhibitor of apoptosis protein
NR5A2	Nuclear receptor subfamily 5 group A member 2
HNF1B	HNF1 homeobox B
FOXF1	Forkhead box F1
GATA4	GATA binding protein 4
VEGFA	Vascular endothelial growth factor A
PROX1	Prospero homeobox 1
TNFR2	TNF receptor 2
EBV	Epstein–Barr virus
IL-12	Interleukin-12
TGM2	Transglutaminase 2
TGF-β	Transforming growth factor-β
DMEM	Dulbecco’s modified Eagle’s medium
PFU	Plaque-forming unit
NC	Negative control
TCID_50_	50% tissue culture infection doses
NOD	Nucleotide.bindingoligomerization domain
Jak-STAT	Janus kinase-Signal transducers and activators of transcription
PPAR	Peroxisome proliferators-activated receptor
NF-κB	Nuclear factor κ-light-chain-enhancer of activated B cells
IκB	Inhibitor of NF-κB
PMSF	Phenylmethanesulfonyl fluoride

## References

[1] Chen S., Hao H., Liu Q., Wang R., Zhang P., Wang X., Du E., Yang Z. (2013). Phylogenetic and pathogenic analyses of two virulent Newcastle disease viruses isolated from Crested Ibis (Nipponia nippon) in China. Virus Genes.

[2] Ganar K., Das M., Sinha S., Kumar S. (2014). Newcastle disease virus: Current status and our understanding. Virus Res..

[3] Brown V.R., Bevins S.N. (2017). A review of virulent Newcastle disease viruses in the United States and the role of wild birds in viral persistence and spread. Vet. Res..

[4] Miller P.J., Kim L.M., Ip H.S., Afonso C.L. (2009). Evolutionary dynamics of Newcastle disease virus. Virology.

[5] Dortmans J.C., Koch G., Rottier P.J., Peeters B.P. (2011). Virulence of Newcastle disease virus: What is known so far?. Vet. Res..

[6] Choi K.S., Kye S.J., Kim J.Y., To T.L., Nguyen D.T., Lee Y.J., Choi J.G., Kang H.M., Kim K.I., Song B.M. (2014). Molecular epidemiology of Newcastle disease viruses in Vietnam. Trop. Anim. Health Prod..

[7] Capraro G.A., Johnson J.B., Kock N.D., Parks G.D. (2008). Virus growth and antibody responses following respiratory tract infection of ferrets and mice with WT and P/V mutants of the paramyxovirus Simian Virus 5. Virology.

[8] Qiu X., Fu Q., Meng C., Yu S., Zhan Y., Dong L., Song C., Sun Y., Tan L., Hu S. (2016). Newcastle Disease Virus V Protein Targets Phosphorylated STAT1 to Block IFN-I Signaling. PLoS ONE.

[9] Rue C.A., Susta L., Cornax I., Brown C.C., Kapczynski D.R., Suarez D.L., King D.J., Miller P.J., Afonso C.L. (2011). Virulent Newcastle disease virus elicits a strong innate immune response in chickens. J. Gen.Virol..

[10] Chen C., Li H., Xie Q., Shang H., Ji J., Bai S., Cao Y., Ma Y., Bi Y. (2011). Transcriptional profiling of host gene expression in chicken liver tissues infected with oncogenic Marek’s disease virus. J. Gen. Virol..

[11] Glennon N.B., Jabado O., Lo M.K., Shaw M.L. (2015). Transcriptome Profiling of the Virus-Induced Innate Immune Response in Pteropus vampyrus and Its Attenuation by Nipah Virus Interferon Antagonist Functions. J. Virol..

[12] Wang Z., Gerstein M., Snyder M. (2009). RNA-Seq: A revolutionary tool for transcriptomics. Nat. Rev. Genet..

[13] Mardis E.R. (2008). The impact of next-generation sequencing technology on genetics. Trends Genet. TIG.

[14] Ha M., Kim V.N. (2014). Regulation of microRNA biogenesis. Nat. Rev. Mol. Cell Biol..

[15] Bruscella P., Bottini S., Baudesson C., Pawlotsky J.M., Feray C., Trabucchi M. (2017). Viruses and miRNAs: More Friends than Foes. Front. Microbiol..

[16] Chen J., Du G., Wang Y., Shi L., Mi J., Tang G. (2017). Integrative analysis of mRNA and miRNA expression profiles in oral lichen planus: Preliminary results. Oral Surg. Oral Med. Oral Pathol. Oral Radiol..

[17] Krutzfeldt J., Stoffel M. (2006). MicroRNAs: A new class of regulatory genes affecting metabolism. Cell Metab..

[18] Bartel D.P. (2004). MicroRNAs: Genomics, biogenesis, mechanism, and function. Cell.

[19] Lin J., Xia J., Chen Y.T., Zhang K.Y., Zeng Y., Yang Q. (2017). H9N2 avian influenza virus enhances the immune responses of BMDCs by down-regulating miR29c. Vaccine.

[20] Ouyang W., Wang Y.S., Du X.N., Liu H.J., Zhang H.B. (2015). gga-miR-9* inhibits IFN production in antiviral innate immunity by targeting interferon regulatory factor 2 to promote IBDV replication. Vet. Microbiol..

[21] Chi J.Q., Teng M., Yu Z.H., Xu H., Su J.W., Zhao P., Xing G.X., Liang H.D., Deng R.G., Qu L.H. (2015). Marek’s disease virus-encoded analog of microRNA-155 activates the oncogene c-Myc by targeting LTBP1 and suppressing the TGF-β signaling pathway. Virology.

[22] Conrad K.D., Giering F., Erfurth C., Neumann A., Fehr C., Meister G., Niepmann M. (2013). microRNA-122 Dependent Binding of Ago2 Protein to Hepatitis C Virus RNA Is Associated with Enhanced RNA Stability and Translation Stimulation. PLoS ONE.

[23] Trobaugh D.W., Klimstra W.B. (2017). MicroRNA Regulation of RNA Virus Replication and Pathogenesis. Trends Mol. Med..

[24] Thibault P.A., Huys A., Amador-Canizares Y., Gailius J.E., Pinel D.E., Wilson J.A. (2015). Regulation of Hepatitis C Virus Genome Replication by Xrn1 and MicroRNA-122 Binding to Individual Sites in the 5’ Untranslated Region. J. Virol..

[25] Ingle H., Kumar S., Raut A.A., Mishra A., Kulkarni D.D., Kameyama T., Takaoka A., Akira S., Kumar H. (2015). The microRNA miR-485 targets host and influenza virus transcripts to regulate antiviral immunity and restrict viral replication. Sci. Signal.

[26] Bandiera S., Pernot S., El Saghire H., Durand S.C., Thumann C., Crouchet E., Ye T., Fofana I., Oudot M.A., Barths J. (2016). Hepatitis C Virus-Induced Upregulation of MicroRNA miR-146a-5p in Hepatocytes Promotes Viral Infection and Deregulates Metabolic Pathways Associated with Liver Disease Pathogenesis. J. Virol..

[27] Munir S., Sharma J.M., Kapur V. (2005). Transcriptional response of avian cells to infection with Newcastle disease virus. Virus Res..

[28] Balasubramaniam V.R., Wai T.H., Omar A.R., Othman I., Hassan S.S. (2012). Cellular transcripts of chicken brain tissues in response to H5N1 and Newcastle disease virus infection. Virol. J..

[29] Hu Z., Hu J., Hu S., Song Q., Ding P., Zhu J., Liu X., Wang X., Liu X. (2015). High levels of virus replication and an intense inflammatory response contribute to the severe pathology in lymphoid tissues caused by Newcastle disease virus genotype VIId. Arch. Virol..

[30] Krutzfeldt J., Poy M.N., Stoffel M. (2006). Strategies to determine the biological function of microRNAs. Nat. Genet..

[31] Zhang S., Xie Y., Cao H., Wang H. (2017). Common microRNA-mRNA interactions exist among distinct porcine iPSC lines independent of their metastable pluripotent states. Cell Death Dis..

[32] Zhang Y., Jing J., Li X., Wang J., Feng X., Cao R., Chen P. (2015). Integration analysis of miRNA and mRNA expression profiles in swine testis cells infected with Japanese encephalitis virus. Infect. Genet. Evol. J. Mol. Epidemiol. Evolut. Genet. Infecti. Dis..

[33] Fiorucci G., Chiantore M.V., Mangino G., Romeo G. (2015). MicroRNAs in virus-induced tumorigenesis and IFN system. Cytokine Growth Factor Rev..

[34] Thakar J., Hartmann B.M., Marjanovic N., Sealfon S.C., Kleinstein S.H. (2015). Comparative analysis of anti-viral transcriptomics reveals novel effects of influenza immune antagonism. BMC Immunol..

[35] Trejo-Avila L.M., Elizondo-Gonzalez R., Rodriguez-Santillan P., Aguilar-Briseno J.A., Ricque-Marie D., Rodriguez-Padilla C., Cruz-Suarez L.E. (2016). Innocuity and anti-Newcastle-virus-activity of Cladosiphon okamuranus fucoidan in chicken embryos. Poult. Sci..

[36] Bolha L., Bencina D., Cizelj I., Oven I., Slavec B., Rojs O.Z., Narat M. (2013). Effect of Mycoplasma synoviae and lentogenic Newcastle disease virus coinfection on cytokine and chemokine gene expression in chicken embryos. Poul. Sci..

[37] Primo M.N., Bak R.O., Schibler B., Mikkelsen J.G. (2012). Regulation of pro-inflammatory cytokines TNFα and IL24 by microRNA-203 in primary keratinocytes. Cytokine.

[38] Miao L., Liu K., Xie M., Xing Y., Xi T. (2014). miR-375 inhibits *Helicobacter pylori*-induced gastric carcinogenesis by blocking JAK2-STAT3 signaling. Cancer Immunol. Immunother. CII.

[39] Shinohara M.L., Lu L., Bu J., Werneck M.B., Kobayashi K.S., Glimcher L.H., Cantor H. (2006). Osteopontin expression is essential for interferon-α production by plasmacytoid dendritic cells. Nat. Immunol..

[40] Rani A., Murphy J.J. (2016). STAT5 in Cancer and Immunity. J. Interferon Cytokine Res..

[41] Villarino A., Laurence A., Robinson G.W., Bonelli M., Dema B., Afzali B., Shih H.Y., Sun H.W., Brooks S.R., Hennighausen L. (2016). Signal transducer and activator of transcription 5 (STAT5) paralog dose governs T cell effector and regulatory functions. eLife.

[42] Beug S.T., Cheung H.H., LaCasse E.C., Korneluk R.G. (2012). Modulation of immune signalling by inhibitors of apoptosis. Trends Immunol..

[43] Anderton H., Rickard J.A., Varigos G.A., Lalaoui N., Silke J. (2017). Inhibitor of Apoptosis Proteins (IAPs) Limit RIPK1-Mediated Skin Inflammation. J. Investig. Dermatol..

[44] Mellett M., Atzei P., Bergin R., Horgan A., Floss T., Wurst W., Callanan J.J., Moynagh P.N. (2015). Orphan receptor IL-17RD regulates Toll-like receptor signalling via SEFIR/TIR interactions. Nat. Commun..

[45] Yang S., Wang Y., Mei K., Zhang S., Sun X., Ren F., Liu S., Yang Z., Wang X., Qin Z. (2015). Tumor necrosis factor receptor 2 (TNFR2).interleukin-17 receptor D (IL-17RD) heteromerization reveals a novel mechanism for NF-κB activation. J. Biol. Chem..

[46] Moriya N., Shibasaki S., Karasaki M., Iwasaki T. (2017). The Impact of MicroRNA-223-3p on IL-17 Receptor D Expression in Synovial Cells. PLoS ONE.

[47] Chang Z., Wang Y., Bian L., Liu Q., Long J.E. (2017). Enterovirus 71 antagonizes the antiviral activity of host STAT3 and IL-6R with partial dependence on virus-induced miR-124. J. Gen. Virol..

[48] Hu Z., Luo D., Wang D., Ma L., Zhao Y., Li L. (2017). IL-17 Activates the IL-6/STAT3 Signal Pathway in the Proliferation of Hepatitis B Virus-Related Hepatocellular Carcinoma. Cell. Physiol. Biochem..

[49] Skinner C.M., Ivanov N.S., Barr S.A., Chen Y., Skalsky R.L. (2017). An Epstein-Barr Virus MicroRNA Blocks Interleukin-1 (IL-1) Signaling by Targeting IL-1 Receptor 1. J. Virol..

[50] Zhou M., Chen J., Zhou L., Chen W., Ding G., Cao L. (2014). Pancreatic cancer derived exosomes regulate the expression of TLR4 in dendritic cells via miR-203. Cell. Immunol..

[51] Tovar-Vidales T., Clark A.F., Wordinger R.J. (2011). Focus on molecules: Transglutaminase 2. Exp. Eye Res..

[52] Kim J.H., Hong J.M., Jeong E.M., Lee W.J., Kim H.R., Kang J.S., Kim I.G., Hwang Y.I. (2014). Lack of transglutaminase 2 diminished T-cell responses in mice. Immunology.

[53] Klock C., Diraimondo T.R., Khosla C. (2012). Role of transglutaminase 2 in celiac disease pathogenesis. Semin. Immunopathol..

[54] Nunes I., Gleizes P.E., Metz C.N., Rifkin D.B. (1997). Latent transforming growth factor-β binding protein domains involved in activation and transglutaminase-dependent cross-linking of latent transforming growth factor-β. J. Cell Biol..

[55] Tkach M., Thery C. (2016). Communication by Extracellular Vesicles: Where We Are and Where We Need to Go. Cell.

[56] Holm C.K., Rahbek S.H., Gad H.H., Bak R.O., Jakobsen M.R., Jiang Z., Hansen A.L., Jensen S.K., Sun C., Thomsen M.K. (2016). Influenza A virus targets a cGAS-independent STING pathway that controls enveloped RNA viruses. Nat. Commun..

[57] Anders S., Pyl P.T., Huber W. (2015). HTSeq--a Python framework to work with high-throughput sequencing data. Bioinformatics.

[58] Trapnell C., Williams B.A., Pertea G., Mortazavi A., Kwan G., van Baren M.J., Salzberg S.L., Wold B.J., Pachter L. (2010). Transcript assembly and quantification by RNA-Seq reveals unannotated transcripts and isoform switching during cell differentiation. Nat. Biotechnol..

[59] Wang L.K., Feng Z.X., Wang X., Wang X.W., Zhang X.G. (2010). DEGseq: An R package for identifying differentially expressed genes from RNA-seq data. Bioinformatics.

[60] Xie C., Mao X., Huang J., Ding Y., Wu J., Dong S., Kong L., Gao G., Li C.Y., Wei L. (2011). KOBAS 2.0: A web server for annotation and identification of enriched pathways and diseases. Nucleic Acids Res..

[61] Livak K.J., Schmittgen T.D. (2001). Analysis of relative gene expression data using real-time quantitative PCR and the 2(-Delta Delta C(T)) Method. Methods.

[62] Liu A.L., Li Y.F., Qi W., Ma X.L., Yu K.X., Huang B., Liao M., Li F., Pan J., Song M.X. (2015). Comparative analysis of selected innate immune-related genes following infection of immortal DF-1 cells with highly pathogenic (H5N1) and low pathogenic (H9N2) avian influenza viruses. Virus Genes.

[63] Dong Y.F., Soung do Y., Schwarz E.M., O’Keefe R.J., Drissi H. (2006). Wnt induction of chondrocyte hypertrophy through the Runx2 transcription factor. J. Cell. Physiol..

[64] Xiao S.Q., Wang X., Ni H.B., Li N., Zhang A.K., Liu H.L., Pu F.X., Xu L.L., Gao J.M., Zhao Q. (2015). MicroRNA miR-24-3p Promotes Porcine Reproductive and Respiratory Syndrome Virus Replication through Suppression of Heme Oxygenase-1 Expression. J. Virol..

[65] Pizzi M. (1950). Sampling variation of the fifty percent end-point, determined by the Reed-Muench (Behrens) method. Hum. Biol..

