# Supplementary figures and images for: Common microRNA–mRNA Interactions in Different Newcastle Disease Virus-Infected Chicken Embryonic Visceral Tissues
Source: Int J Mol Sci. 2018 Apr 25;19(5):1291. doi: 10.3390/ijms19051291 (PMC5983721; doi:10.3390/ijms19051291)

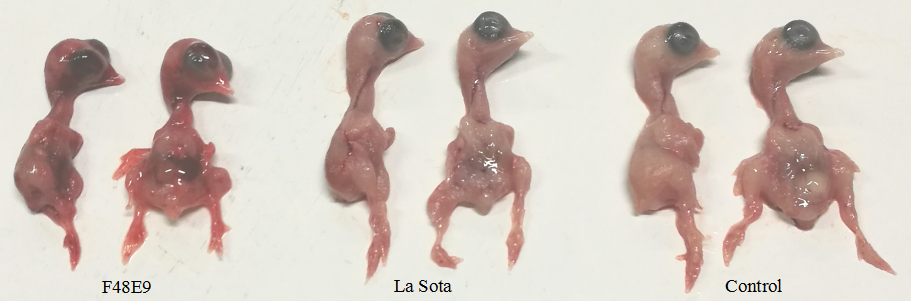

Supplement: Supplementary file 1 [file ijms-19-01291-s001.zip › Supplementary Files/Figure S1.tif]
